# Diet Soft Drink Consumption is Associated with the Metabolic Syndrome: A Two Sample Comparison

**DOI:** 10.3390/nu7053569

**Published:** 2015-05-13

**Authors:** Georgina Crichton, Ala’a Alkerwi, Merrrill Elias

**Affiliations:** 1 Alliance for Research in Exercise, Nutrition and Activity (ARENA), Sansom Institute for Health Research, University of South Australia, Adelaide 5001, Australia; 2 Luxembourg Health Institute (L.I.H.) (formerly Centre de Recherche Public Santé, Centre d’Etudes en Santé), L-1445, Grand-Duchy of Luxembourg; E-Mail: Alaa.Alkerwi@lih.lu; 3 Department of Psychology, University of Maine, Orono, ME 04469, USA; E-Mail: mfelias@maine.edu; 4 Graduate School of Biomedical Sciences and Engineering, University of Maine, Orono, ME 04469, USA

**Keywords:** soft drink, diet soft drink, metabolic syndrome, international comparison

## Abstract

Comparative analyses of soft drink intakes in samples from the United States and Europe, and assessed intakes in relation to prevalence of metabolic syndrome (MetS) and its individual components are currently lacking. We used data collected on cardiovascular health and dietary intakes in participants from two cross-sectional studies: the Maine-Syracuse Longitudinal Study (MSLS), conducted in Central New York, USA in 2001–2006 (*n* = 803), and the Observation of Cardiovascular Risk Factors in Luxembourg Study (ORISCAV-LUX), conducted in 2007–2009 (*n* = 1323). Odds ratios for MetS were estimated according to type and quantity of soft drink consumption, adjusting for demographic, lifestyle and dietary factors, in both studies. In both studies, individuals who consumed at least one soft drink per day had a higher prevalence of MetS, than non-consumers. This was most evident for consumers of diet soft drinks, consistent across both studies. Diet soft drink intakes were also positively associated with waist circumference and fasting plasma glucose in both studies. Despite quite different consumption patterns of diet *versus* regular soft drinks in the two studies, findings from both support the notion that diet soft drinks are associated with a higher prevalence of MetS.

## 1. Introduction

Metabolic syndrome (MetS) is characterised by a clustering of cardiometabolic risk factors within an individual, namely abdominal obesity, hypertension, and dyslipidemia [1]. Having MetS increases the risk for cardiovascular disease (CVD), coronary heart disease, stroke, and type 2 diabetes mellitus [2,3]. Modifiable lifestyle factors, such as diet, are a primary contributor to both the development and subsequent course of MetS [4]. The average intakes of “added sugars” in the US, estimated from the National Health and Nutrition Examination Survey (NHANES), is 22 teaspoons per day [5], of which soft drinks and fruit drinks provide more than 40% [6]. Consumption of soft drinks is increasing amongst men, women and children in the US and also in Europe [7,8,9,10]. This may include both sugar-sweetened ‘regular’, and artificially sweetened ‘diet’ soft drinks. Substantial epidemiological evidence for the association between high intakes of sugar-sweetened beverages and risk for obesity, type 2 diabetes, and CVD exists [11,12,13,14,15]. While replacing sugar-sweetened soft drinks with diet, sugar-free, or artificially-sweetened beverages may be used to reduce sugar intake, recent research has demonstrated associations between diet soft drink consumption and adverse cardiometabolic outcomes [16,17,18,19].

Diet soft drink intakes have been positively associated with MetS prevalence in the Framingham Offspring Study [16], and with greater relative risk of incident MetS in the Multi-Ethnic Study of Atherosclerosis (MESA) [19], and in the Atherosclerosis Risk in Communities Study (ARIC) [18].

These studies have all been conducted in the US [16,18,19]. Less evidence for detrimental health effects associated with high soda consumption has emerged from Europe. One study has shown a positive association between sugar-sweetened beverages and six-year risk of MetS in a Mediterranean cohort of university graduates [20]. The Oslo Health Study showed a positive association between a diet index reflecting a high intake of soft drinks (including sugar-free) and a low intake of fruit and vegetables with components of MetS [21]. Further research in European samples is warranted. 

The objective of the present study was to evaluate soft drink intakes from two cross-sectional study samples in central New York (Maine-Syracuse Longitudinal Study, MSLS, USA), and Luxembourg (Observation of Cardiovascular Risk Factors in Luxembourg, ORISCAV-LUX), and to assess intakes in relation to the prevalence of MetS and its individual components. Comparisons between two male cohorts from America and Italy have been made in relation to cardiovascular health risk factors and alcohol intake [22]. To our knowledge, no such comparisons have been made for soft drink consumption, in relation to cardiovascular health, and MetS in particular. 

In addition, we aimed to determine whether the prevalence of MetS differed according to the type of soft drink consumed (regular *versus* diet). In our past examination of the health and lifestyle habits in these two studies, we found that the ORISCAV-LUX participants had healthier diets and lower levels of obesity, hypertension, diabetes and CVD than those in MSLS [23]. The following hypotheses were therefore advanced: (1) overall soft drink consumption would be higher in the MSLS than in ORISCAV-LUX; (2) positive relations would be found between both regular and diet soft drink intakes and MetS prevalence in both studies; and (3) modestly stronger relations would be observed in the MSLS.

## 2. Methods

### 2.1. Study Design and Sample

The total sample comprised from two studies, the MSLS, and the ORISCAV-LUX, included 2126 individuals, 803 from MSLS and 1323 from ORISCAV-LUX. Further details related to the methods of sampling for both studies appear below and in numerous publications [24,25,26,27].

#### 2.1.1. Participants in MSLS (USA)

The MSLS is a longitudinal, community-based study of aging, cardiovascular risk factors and cognitive functioning in adults, aged 23–98 years [26,27,28,29]. The MSLS was conducted in Syracuse, New York (NY), USA and its catchment area (Central NY). At initial recruitment (1975), the sole exclusions were institutionalized people, diagnosed alcoholism and psychiatric disorder. The data for the present cross-sectional study were taken from subjects returning for the sixth (2001–2006) study wave when dietary intake measures were first obtained and data on objectively measured cardiovascular risk factors were available. Beginning with a sample of 1049 individuals, participants were excluded from the present analysis for the following reasons: missing data on diet or components of MetS (*n* = 169), acute stroke (*n* = 28), probable dementia (*n* = 8), undergoing hemo-dialysis (*n* = 5), inability to read English (*n* = 1), and alcohol abuse after baseline (*n* = 1), leaving 803 participants. 

The University of Maine Institutional Review Board approved this study and informed consent was obtained from all participants.

#### 2.1.2. Participants in ORISCAV-LUX (Luxembourg)

ORISCAV-LUX was a nationwide, cross-sectional study on the prevalence of cardiovascular risk factors among the adult population of Luxembourg, aged 18–69 years, conducted in 2007–2009. Exclusions were pregnancy (*n* = 21), serious mental and/or physical handicap (*n* = 5), prisoners (*n* = 1), people outside the determined age range (*n* = 2) and those deceased before recruitment (*n* = 5) [25]. A representative random sample of 1432 individuals, stratified by sex, age, and district of residence completed the recruitment procedure [24,25]. After data cleaning, the total ORISCAV-LUX sample comprised 1323 individuals.

The study was approved by the National Research Ethics Committee and the National Commission for Private Data Protection, and all participants gave informed written consent.

### 2.2. Procedure

#### 2.2.1. Dietary Assessment

In the MSLS, dietary intake was assessed using the food frequency questionnaire (FFQ) component of the Nutrition and Health Questionnaire [30,31,32]. Its acceptable validity has been demonstrated by comparison with dietary recall, protein excretion and total energy expenditure data [30]. The dietary component questions participants about their frequency of consumption of 37 foods and beverages. Participants are required to stipulate their frequency of consumption, with six response options: “never”, “seldom”, “once a week”, “2–4 times/week”, “5–6 times/week” and “once or more/day”. For soft drinks, participants were asked to report how many glasses/cans of “diet” carbonated soft drinks and “regular” carbonated soft drinks they consumed daily. These two intakes were summed to give total soft drink intake per day. Portion or serving sizes were not stipulated; therefore total energy was estimated in the following manner: the median score within each response option was used to estimate total intakes per week; for example, two to four times per week was estimated at three. The mean number of times each food was consumed on a weekly and then daily basis was calculated for all foods. Individual foods were categorized into six major food groups (grains, fruits, vegetables, protein foods, dairy foods, and fats/sweets/other). Total energy was therefore estimated by summing the number of servings per day of all foods and beverages [33].

In ORISCAV-LUX, dietary intake was assessed using a validated, semi-quantified FFQ, which assessed the frequency of consumption of 134 items over the previous three months [34,35]. Participants were asked how frequently they consumed one standardized portion of each food. Both diet and regular soft drinks were included in the FFQ, and for beverages, there were five frequency response categories: “never or rarely”, “1–3 times/month”, “1–2 times/week”, “3–5 times/week”, and “every day”. Participants were also required to indicate the total quantity (in mL) of drink they consumed each time. This enabled the calculation of daily intakes of diet, regular, and total soft drinks (in servings per day, 330 mL equating to one serving). Energy and nutrient intake data, including alcohol (g/day) and total energy intake (Kcal/day), were compiled.

#### 2.2.2. Lifestyle and Heath Data

Participants in both studies underwent physical and anthropometric measurements, and blood tests. Standardized protocols for data collection were used. Body weight, height, body mass index (BMI), waist circumference, and blood pressure (BP) measures were assessed as described previously for both studies [24,25,26,27,28,36]. Standard assay methods were employed [26,36] to obtain fasting plasma glucose, serum triglycerides, and HDL-cholesterol, as well as low-density lipoprotein (LDL)-cholesterol and total cholesterol (all mg/dL). 

All participants completed self-administered questionnaires to gain information on demographic and socioeconomic characteristics, including age, sex, education (years), smoking (cigarettes/day), and physical activity (minutes/day). In the MSLS, physical activity was measured with the Nurses’ Health Study (NHS) Activity Questionnaire [37]. Smoking status was based on self-report from the Nutrition and Health Questionnaire [30]. In ORISCAV-LUX, physical activity was measured using the short format International Physical Activity Questionnaire (IPAQ) [38]. Detailed data regarding smoking were obtained from the health questionnaire. 

#### 2.2.3. Definition of MetS

MetS (and components) was defined by National Cholesterol Education Program Adult Treatment Panel III criteria. MetS was defined as present if three out of five risk factors were present: waist circumference ≥ 88 cm for women or 102 cm for men; fasting glucose ≥ 100 mg/dL (or drug treatment); blood pressure ≥135/85 mmHg or treatment for hypertension; serum triglycerides ≥150 mg/dL (or drug treatment); and high density lipoprotein (HDL)-cholesterol ≤ 40 mg/dL in men or 50 mg/dL in women [39].

### 2.3. Statistical Analysis

Participant characteristics in each study were compared according to soft drink consumption: non-consumer, and three consumer groups (diet, regular, or mixed diet/regular). A consumer was defined as someone who reported consuming any type of soft drink. For continuous variables, analysis of variance (ANOVA) was used to compare the four groups in terms of demographics, health factors, and dietary variables, with Bonferroni adjustment for multiple comparisons. For categorical health-related variables, Chi square tests were performed. For the primary analyses, logistic regression analyses were used to compare the prevalence of MetS in participants who consumed soft drink (one per day, two or more per day), compared to non-consumers (referent group). This was performed for diet, regular, and total soft drinks. The same analyses were performed for the MSLS (*n* = 803) and for ORICAV-LUX (*n* = 1323). The following three multivariable regression models were used: 

Model 1: adjusted for demographic and lifestyle factors, including age, sex, education, smoking, and physical activity.

Model 2: Model 1 plus adjusted for dietary factors including intakes of alcohol, vegetables, fruit, grains and meat. 

Model 3: Models 1 and 2 plus adjusted for total energy intake.

When assessing relations between diet soft drink and MetS prevalence, regular soft drink intake (servings/day) was included in Models 2 and 3; similarly diet soft drink intake was added to Models 2 and 3 when assessing associations between regular soft drink intake and MetS. 

Finally, multiple linear regression analyses were used to evaluate relations between soft drink consumption and each of the individual components of MetS, as continuous variables (waist circumference, systolic and diastolic BP, HDL-cholesterol, triglycerides, and fasting plasma glucose), in each study. The same covariable sets were used as for the logistic regression analyses (see above).

All statistical analyses were performed with PASW for Windows^®^ version 21.0 software (formerly SPSS Statistics Inc. Chicago, IL, USA); *p* < 0.05 was considered statistically significant.

## 3. Results

### 3.1. Participant Characteristics and Soft Drink Consumption

Table 1 shows the demographic variables, MetS components, dietary intakes and other health-related variables for MSLS and ORISCAV-LUX participants, according to the type of soft drink consumed (non-consumer, diet only, regular only, or mix diet/regular). Supplementary Table S1 shows these data for the total samples from each study.

**Table 1:** Study sample characteristics according to daily soft drink consumption in ORISCAV-LUX and MSLS studies.

Characteristic	MSLS, *n* = 803				ORISCAV-LUX, *n* = 1323			
	Soft Drink Consumption				Soft Drink Consumption			
	Non-consumer	Diet only	Regular only	Diet and regular	Non-consumer	Diet only	Regular only	Diet and regular
*n* (%)	460 (57.3)	192 (23.9)	130 (16.2)	21 (2.6)	525 (39.7)	139 (10.5)	484 (36.6)	175 (13.2)
Age (years)	64.5 ± 12.9	59.3 ± 11.2 ^1^	55.6 ± 12.2 ^1^	61.7 ± 14.8	49.2 ± 12.2	45.5 ± 12.8 ^1^	40.9 ± 12.6 ^1^	38.7 ± 11.9 ^1^
Sex (% male)	36.3	36.5	56.2	57.1	40.6	36.0	58.8	54.3
Mean no. soft drinks/day	0	1.7 ± 1.2 ^1^	1.8 ± 1.8 ^1^	3.1 ± 1.9 ^1,2,3^	0	0.8 ± 1.1 ^1^	0.8 ± 1.2 ^1,4^	1.1 ± 1.7 ^1^
Physical activity (mins/day)	35 ± 47	40 ± 49	39 ± 57	34 ± 43	100 ± 122	116 ± 131	117 ± 149	106 ± 124
Smoking (cigs/day)	0.7 ± 3.7	1.2 ± 5.7	4.0 ± 8.5 ^1,2,4^	0	2.1 ± 6.3	1.7 ± 5.3	4.1 ± 8.1 ^1,2,4^	2.1 ± 6.4
MetS (% within each group)	38.7	49.0	55.4	42.9	26.9	33.8	24.6	22.3
Systolic BP (mmHg)	131 ± 22	131 ± 23	131 ± 22	138 ± 26	132 ± 18	134 ± 20	129 ± 17 ^1,2^	128 ± 17 ^2^
Diastolic BP (mmHg)	70 ± 10	70 ± 9.2	71 ± 10	71 ± 13	83 ± 10	83 ± 11	82 ± 11	81 ± 11
Waist circumference (cm)	92 ± 15	96 ± 14 ^1^	102 ± 15 ^1,2^	101 ± 25	90 ± 14	93 ± 15	89 ± 13 ^2^	90 ± 15
Total cholesterol (mg/dL)	204 ± 38	199 ± 41	204 ± 45	194 ± 41	207 ± 40	199 ± 37	199 ± 42 ^1^	196 ± 39 ^1^
HDL cholesterol (mg/dL)	56 ± 16	54 ± 16	45 ± 11 ^1,2,4^	57 ± 17	64 ± 18	62 ± 15	60 ± 16 ^1^	59 ± 17 ^1^
LDL cholesterol (mg/dL)	122 ± 32	118 ± 33	125 ± 38	118 ± 30	128 ± 35	121 ± 36	123 ± 36	120 ± 33
Fasting plasma glucose (mg/dL)	97 ± 28	101 ± 29	100 ± 20	102 ± 46	97 ± 22	98 ± 20	94 ± 16	92 ± 10 ^1,2^
Triglycerides (mg/dL)	135 ± 90	140 ± 98	189 ± 176 ^1,2,4^	118 ± 64	110 ± 87	119 ± 80	118 ± 94	123 ± 126
BMI (kg/m^2^)	28.1 ± 5.4	30.2 ± 5.6 ^1^	31.7 ± 7.4 ^1^	30.7 ± 9.9 *	26.5 ± 5.0	28.0 ± 5.3 ^1,3^	26.0 ± 4.7	27.0 ± 5.2
Diabetes mellitus (%)	9.6	16.1	13.8	14.3	25.6	28.8	20.2	20.6
Hypertension (%)	60.4	60.9	63.1	66.7	45.9	44.6	35.3	29.7
Obesity (%)	30.2	46.8	50.8	42.9	21.0	30.9	20.2	27.4
Dietary variables								
Total energy intake ^a^	14.2 ± 4.2	13.6 ± 3.7	16.5 ± 5.5 ^1,2^	16.9 ± 4.5 ^1,2^	2187 ± 808	2223 ± 875	2627 ± 985 ^1,2^	2688 ± 995 ^1,2^
Vegetables (servings/day)	2.8 ± 1.1	2.8 ± 1.1	2.3 ± 1.1 ^1,2^	2.6 ± 1.0	4.2 ± 2.9	4.2 ± 2.9	3.4 ± 2.5 ^1,2^	3.9 ± 2.6
Fruit (servings/day)	1.7 ± 1.1	1.5 ± 0.9	1.4 ± 1.0 ^1^	1.8 ± 0.9	2.0 ± 2.0	1.7 ± 1.7	1.6 ± 1.9 ^1^	1.8 ± 1.8
Grains (servings/day)	3.6 ± 2.0	3.4 ± 1.8 ^4^	4.0 ± 2.2	4.7 ± 1.6	2.8 ± 1.2	2.5 ± 1.3	2.7 ± 1.1	2.9 ± 1.3 ^2^
Meat (servings/day)	2.0 ± 0.8	2.1 ± 1.0	2.2 ± 1.0	2.0 ± 0.8	1.0 ± 0.6	1.1 ± 0.6	1.3 ± 0.7 ^1,2^	1.4 ± 0.8 ^1,2^
Alcohol (standard drinks/day)	0.6 ± 1.0	0.4 ± 0.7	0.4 ± 1.1	0.4 ± 1.6	0.8 ± 0.7	0.8 ± 0.8	0.8 ± 0.8	0.7 ± 0.8

Values are mean ± SD unless otherwise indicated; ^a^ Total energy intake: in Kcal/day (ORISCAV-LUX) and total serves/day all food groups (MSLS); ^1−4^ superscript numbers indicate significant differences (*p* < 0.05) between groups (ANOVA): ^1^ value significantly different from non-consumer group; ^2^ value significantly different from “diet” only group; ^3^ significantly different from “regular” only group; ^4^ significantly different from “diet and regular” group.

A greater proportion of participants in ORICAV-LUX study consumed soft drinks (60% of participants), compared with the MSLS study (43% of participants). Consumption of diet soft drinks was higher in the MSLS (24% of paticipants), compared with 11% in ORISCAV-LUX. More subjects consumed a mix of diet and regular drinks in ORISCAV-LUX than in the MSLS. However, of those consuming soft drinks, the mean number of drinks consumed per day was higher in the MSLS (two servings/day for diet and regular drinks) than in the ORISCAV-LUX study (one serving/day for both drink types). 

In both studies, BMIs were significantly higher in diet soft drink consumers than in non-consumers (both *p* < 0.05). Waist circumference was significantly higher in diet soft drink consumers than in non-consumers in MSLS (*p* < 0.05). From a dietary perspective, higher intakes of total energy, and lower intakes of fruit and vegetables were observed in regular soft drink consumers in both studies than in non-consumers (all *p* < 0.05).

### 3.2. Soft Drink Consumption and Prevalence of MetS in MSLS

Individuals in the MSLS who consumed at least one soft drink per day (regular or diet) had 2.4-fold higher odds of having MetS compared with those who consumed none, after full adjustment for dietary factors and total energy intake (Odds Ratio, OR: 2.4, 95% CI: 1.5-3.9; Table 2). This increased risk was also significant for those who consumed two or more drinks per day (OR: 2.1, 95% CI: 1.2–3.8, full adjustment).

When assessed separately, diet soft drink intakes were significantly associated with odds of having MetS (all models). In the fully adjusted model, those who consumed at least one diet soft drink per day had a significantly higher adjusted prevalence of MetS relative to non-consumers (OR: 2.2, 95% CI: 1.3–3.7). Those who consumed one daily regular soft drink also had higher odds of having MetS (OR: 1.9, 95% CI: 1.1–3.5), however this association was no longer significant with the addition of total energy intake (Table 2).

### 3.3. Soft Drink Consumption and Prevalence of MetS in ORISCAV-LUX

Individuals in the ORISCAV-LUX study who consumed at least one soft drink per day (regular or diet) had two-fold higher odds of having MetS compared to those who consumed none, with full adjustment for potential confounders (OR: 2.0, 95% CI: 1.4–2.8; Table 3). This increased risk was similarly significant for those who consumed two or more drinks per day (OR: 2.1, 95% CI: 1.1–4.0, full adjustment). 

A higher prevalence of MetS was observed in those who consumed diet soft drinks (Model 1), and this remained significant with adjustment for dietary variables and total energy intake for those who consumed at least two servings per day (Model 3: OR: 3.9, 95% CI: 1.5–10.3). A higher prevalence of MetS was also evident in those who consumed at least one regular soft drink per day, relative to non-consumers (OR: 1.7, 95% CI: 1.2–2.4, full adjustment; Table 3).

**Table 2:** Cross-sectional relationships between soft drink consumption with prevalence of MetS in MSLS (*N* = 803).

Soft Drink Consumption (Servings/day)	*n* (%) of Sample	% with MetS within each Group	MSLS, USA, *n* = 803					
			Model 1		Model 2		Model 3	
			OR	95% CI	OR	95% CI	OR	95% CI
**Total (regular, diet, or both)**								
None	460 (57.3)	178 (38.7)	1 (Reference group)		1 (Reference group)		1 (Reference group)	
1 per day	201 (25.0)	103 (51.2)	2.5 ^***^	1.5–3.9	2.4 ^***^	1.5–3.9	2.4 ^***^	1.5–3.9
2 or more per day	142 (17.7)	72 (50.7)	2.5 ^**^	1.4–4.3	2.2 ^**^	1.2–3.9	2.1 ^*^	1.2–3.8
**Diet ^a^**								
None	590 (73.5)	250 (42.4)	1 (Reference group)		1 (Reference group)		1 (Reference group)	
1 per day	136 (16.9)	67 (49.3)	1.9 ^*^	1.2–3.2	2.2 ^**^	1.3–3.7	2.2 ^**^	1.3–3.7
2 or more per day	77 (9.6)	36 (46.8)	1.9	1.0–3.7	1.7	0.9–3.3	1.8	0.9–3.5
**Regular ^b^**								
None	650 (81.1)	271 (41.7)	1 (Reference group)		1 (Reference group)		1 (Reference group)	
1 per day	92 (11.5)	48 (52.2)	1.8	1.0–3.2	1.9 ^*^	1.1–3.5	1.8	0.9–3.4
2 or more per day	59 (7.4)	33 (55.9)	1.9	0.8–4.5	1.9	0.8–4.6	1.7	0.7–4.5

^*^ *p* < 0.05, ^**^ *p* < 0.01, ^***^ *p* < 0.001; Model 1: adjusted for age, sex, education, smoking (cigarettes/day), physical activity (mins/day); Model 2: adjusted for Model 1 covariates + alcohol (standard drinks/day), total intakes of vegetables, fruit, grains and meat (all servings/day); ^a^ Model 2 and 3: regular soft drinks/day added; ^b^ Model 2 and 3: diet soft drinks/day added; Model 3: adjusted for Model 1 & 2 covariates + total energy (serves/day all food groups); OR = odds ratio; CI = confidence interval.

**Table 3:** Cross-sectional relationships between soft drink consumption with prevalence of MetS in ORISCAV-LUX (*N* = 1323).

Soft Drink Consumption (Servings/day)	% of total Sample	% with MetS within each Group	ORISCAV-LUX, Luxembourg, *n* = 1323					
			Model 1		Model 2		Model 3	
			OR	95% CI	OR	95% CI	OR	95% CI
**Total (regular, diet, or both)**								
None	525 (39.7)	141 (26.9)	1 (Reference group)		1 (Reference group)		1 (Reference group)	
1 per day	685 (51.8)	179 (26.1)	1.7 ^**^	1.2–2.3	1.9 ^***^	1.3–2.6	2.0 ^***^	1.4–2.8
2 or more per day	113 (8.5)	26 (23.0)	1.5	0.8–2.6	1.5	0.8–2.9	2.1 ^*^	1.1–4.0
**Diet ^a^**								
None	1009 (76.3)	260 (25.8)	1 (Reference group)		1 (Reference group)		1 (Reference group)	
1 per day	287 (21.7)	74 (25.8)	1.6 ^*^	1.1–2.2	1.3	0.9–2.0	1.4	0.9–2.0
2 or more per day	27 (2.0)	12 (44.4)	4.5 ^**^	1.8–11.4	3.7 ^**^	1.4–9.8	3.9 ^**^	1.5–10.3
**Regular ^b^**								
None	664 (50.2)	188 (28.3)	1 (Reference group)		1 (Reference group)		1 (Reference group)	
1 per day	577 (43.6)	146 (25.3)	1.4 ^*^	1.0–1.9	1.6 ^**^	1.2–2.3	1.7 ^**^	1.2–2.4
2 or more per day	82 (6.2)	12 (14.6)	0.6	0.3–1.2	0.6	0.3–1.3	0.8	0.3–1.8

^*^ *p* < 0.05, ^**^ *p* < 0.01, ^***^ *p* < 0.001; Model 1: adjusted for age, sex, education, smoking (cigarettes/day), physical activity (mins/day); Model 2: adjusted for Model 1 covariates + alcohol (standard drinks/day), total intakes of vegetables, fruit, grains and meat (all servings/day); ^a^ Model 2 and 3: regular soft drinks/day added; ^b^ Model 2 and 3: diet soft drinks/day added; Model 3: adjusted for Model 1 and 2 covariates + total energy (Kcal/day); OR = odds ratio; CI = confidence interval.

### 3.4. Soft Drink Consumption and Individual Components of MetS

In both studies, diet soft drink consumption was positively associated with waist circumference (both *p* < 0.05, model 2, Table 4). In ORISCV-LUX, diet soft drinks were also positively associated with systolic BP and with fasting plasma glucose levels (both *p* < 0.05). Regular soft drinks were inversely associated with waist circumference in ORISCAV-LUX (*p* = 0.001), but positively associated with waist circumference in MSLS (*p* < 0.001). Sugar-sweetened soft drinks were also positively associated with triglyceride levels in MSLS (*p* < 0.01).

It is possible that age accounted for the fact that the Central NY sample was more overweight and centrally obese than the Luxembourg sample, as the American sample had a greater proportion of older adults. Thus, in additional analyses we equated the distributions in terms of age for the US and Luxembourg samples (to the common age range of 23 to 69 years). In these samples (*n* = 565 in MSLS; *n* = 1223 in ORISV-LUX), BMI and WC were still significantly higher in the Central NY sample than in Luxembourg (see Supplementary Table S2).

### 3.5. Sensitivity Analyses

A number of sensitivity analyses were performed. Firstly, MetS risk was estimated across diet (artificially-sweetened) soft drink consumption categories excluding the participants who also consumed regular (sugar-sweetened) soft drinks. Similarly, analyses were performed across regular soft drink consumption categories excluding participants who also consumed diet soft drinks. The results remained unchanged in both studies. 

We also excluded those being treated for diabetes (*n* = 77 in MSLS; *n* = 37 in ORISCAV-LUX). The results in Table 2 and Table 3 remained unchanged.

A final sensitivity analysis was performed in the ORICAV-LUX sample, excluding those participants who reported dieting at the time of the survey (*n* = 191). The associations between diet soft drink intakes and MetS remained the same. 

**Table 4:** Cross-sectional relationships between soft drink consumption and MetS components in MSLS and ORISCAV-LUX.

MetS Component	Predictor (Soft Drink) ^a^	MSLS, USA, *n* = 803			ORISCAV-LUX, Luxembourg, *n* = 1323		
		b	SE	*p*	b	SE	*p*
Systolic blood pressure (mmHg)	Diet	0.49	0.74	0.5	1.6	0.78	0.036
	Regular	0.46	0.86	0.6	−0.35	0.51	0.5
Diastolic blood pressure (mmHg)	Diet	−0.09	0.36	0.8	0.13	0.55	0.8
	Regular	−0.13	0.42	0.7	−0.30	0.36	0.4
Waist circumference (cm)	Diet	1.2	0.48	0.01	2.0	0.62	0.001
	Regular	2.1	0.56	<0.001	−1.4	0.41	0.001
HDL-cholesterol (mg/dL)	Diet	0.68	0.50	0.2	−0.70	0.81	0.4
	Regular	−0.29	0.59	0.6	−0.39	0.54	0.5
Triglycerides (mg/dL)	Diet	−5.1	4.1	0.2	2.0	5.0	0.7
	Regular	13.4	4.7	0.005	−4.0	3.3	0.2
Fasting glucose (mg/dL)	Diet	0.34	0.86	0.7	1.6	0.79	0.049
	Regular	0.84	1.0	0.4	−0.65	0.53	0.2

Presented data are for the extended model; raw regression coefficients (b) are adjusted for age, sex, education, total daily energy intake, smoking, physical activity, alcohol, total intakes of vegetables, fruit, grains and meat, medication for hypertension, diabetes or dyslipidemia; ^a^ For diet soft drinks: models adjusted for regular soft drinks/day; for regular soft drinks: models adjusted for diet soft drinks/day; HDL = high density lipoprotein.

## 4. Discussion

Consistent with previous research [16,19], we observed similar, significant associations between increasing soft drink consumption and prevalence of MetS in Central NY, USA and in Luxembourg. In particular, associations between diet soft drink intakes and MetS prevalence were observed. Consistent with these findings, diet soft drinks were positively associated with waist circumference.

These findings were consistent in the two studies, despite quite different soft drink intake patterns. Further, MetS prevalence was considerably higher in the US (Central NY) sample (44%) than in the Luxembourg sample (26%). These data are consistent with a previous comparative analyses of the cardiovascular health of two samples from these studies [23]. Based on these previous findings, and contrary to what we expected, more people consumed soft drinks in ORISCAV-LUX than in MSLS, but the quantities consumed were higher in MSLS. Although we hypothesised that associations may be stronger in MSLS than in ORISCAV-LUX, the odds of having MetS associated with total soft drink intakes were similar in both (approximately 2-fold higher odds). Of soft drinks consumers in the US sample, a greater proportion selected diet drinks over regular/sugar-sweetened soft drinks; while the opposite was true in ORISCAV-LUX, *i.e.*, the proportion of those consuming regular soft drinks was three times higher than those consuming diet drinks. Approximately 2% of the ORISCAV-LUX sample consumed two or more diet soft drinks per day, compared with 10% in the MSLS sample. Interestingly, the MSLS sample was more overweight and centrally obese than the Luxembourg one (as measured by both BMI and waist circumference, respectively), despite fewer persons consuming soft drinks. The age difference in the two samples may also help to explain these observations, as the MSLS participants consisted of a greater number of older adults, who typically consume fewer soft drinks than younger people [9]. In individuals who did consume soft drink, the average daily intake of soft drink consumers (any type) in the MSLS sample was over two times greater than the intake of consumers in ORISCAV-LUX (1.8 ± 1.6 servings/day in MSLS, compared to mean 0.8 ± 1.3 servings/day in ORISCAV-LUX). When we equated the distributions of the two samples (to the common age range of 23 to 69 years) in a secondary analysis, the mean number of diet, regular, and total soft drinks consumed per day (amongst soft drink consumers) remained higher in Central NY than in Luxembourg, when adults aged over 69 years were excluded (see Supplementary Table S1).

Other recent studies have demonstrated that higher levels of soft drink consumption (at least daily intakes) are associated with MetS [16,18,19]. In MESA [19], at least daily consumption of diet soda was associated with a 36% greater relative risk of incident MetS, compared with non-consumption. Of the components, waist circumference and high fasting glucose were prospectively associated with diet soda consumption. The present study confirms these findings, at the level of one daily serving. Increasing to two daily servings did not significantly increase the likelihood of having MetS; in both studies proportions of those with MetS were similar regardless of whether one drink per day, or more than one, was consumed. Furthermore, and consistent with MESA, we showed an increasing dose-response pattern in both samples for waist circumference and in ORISCV-LUX for fasting plasma glucose and systolic BP, with both increasing as more diet soft drink was consumed.

A number of mechanisms have been postulated that may explain the findings observed. Firstly, those who consume higher quantities of soft drinks may also have a dietary and/or lifestyle pattern that is not as healthy as those who do limit these drinks. Total energy intakes were higher in those individuals who consumed regular soft drinks compared to those who consumed diet drinks in both samples. Regular soft drink consumers in the studies analysed here (both MSLS and ORISCAV-LUX) consumed fewer fruit and vegetables, more grains and meats, and smoked more cigarettes than diet soft drink consumers. Findings in a previous study suggest people who consume higher amounts of sugar-containing soft drinks may fail to compensate for these ‘liquid calories’ at their next meal, promoting a positive energy balance and weight gain [40]. The energy compensation made for beverages is not equivalent to that made for solid foods, and therefore the energy content of soft drinks can contribute to a cumulative excess of energy over time to produce obesity [41]. The high fructose corn syrup added to regular soft drinks (the primary sweetener in soft drinks) may also contribute to adverse metabolic effects. Less is known about the physiological mechanisms linking high intakes of sugar-free soft drinks with adverse cardiometabolic outcomes. Animal studies have shown that artificial sweeteners, such as aspartame, may reduce the ability of the body to estimate the energy content of foods, leading to increased intake and body weight gain [42]. However, the safety of aspartame, for use as a sweetener and flavour enhancer, has been established [43]. Some researchers have suggested that the high sweetness in artificially sweetened drinks may result in hunger [44] or greater preference for other sweet or energy dense foods [42]. However other studies have failed to show that artificial sweeteners (including both aspartame and saccharin) increase hunger or subsequent food intake [45,46,47]. Other research suggests that positive relationships may be due to confounding or reverse causality [48]. For example, diet soda consumption has been reported as up to three times higher among adult diabetics in the US than non-diabetics [49]. People diagnosed with heart disease or diabetes may therefore actively opt for artificially sweetened drinks.

Indeed reverse causation may explain the present findings between diet soft drink consumption and higher MetS prevalence. It is possible that some current drinkers of diet soft drinks had replaced regular with diet drinks for health reasons, and therefore continued to exhibit adverse disease patterns. This may be particularly true within the Luxembourg sample, where levels of type 2 diabetes, hypertension and obesity (BMI ≥ 30 kg/m^2^) were higher in diet drinkers than in regular soda drinkers. Excluding those being treated for diabetes (both studies) did not however alter the results. Of note in both samples, was the observation that obesity levels in diet soft drink consumers was significantly higher than in non-drinkers (of any type). It is quite plausible that in response to their body weight status, some individuals may have switched from regular to diet drinks, but not given up soft drinks altogether, explaining the higher obesity levels in this group. However, this cannot fully explain the findings as excluding those who were dieting in the Luxembourg sample did not alter the significant positive associations between diet soft drink consumption and MetS.

### Strengths and Weaknesses

Strengths of the analysis include detailed information in both MSLS and ORISCAV-LUX on diet, cardiometabolic health, and additional covariates in adults. This is the first study that we are aware of to compare relationships between soft drink consumption and MetS in two studies from two different countries. Cross-country comparisons are important to provide insights into the social determinants of dietary habits and health [50].

There are several study limitations. The ORISCAV-LUX was a nationwide, population-based study, whereas MSLS was a community-based sample restricted to Central NY, USA. A broader American sample would enable us to see if there are similar or differing trends in other parts of the US. The cross-sectional nature of both studies prohibits any conclusions with regard to causality. Soft drink intakes and other dietary data were based on participant self-report and the same food questionnaires were not used in both studies. The FFQ used in ORISCAV-LUX was semi-quantitative, with participants reporting frequency of servings and stipulating their serving size in mL. The MSLS participants reported their intakes in terms of glasses or cans per day. The inherent measurement error associated with the use of FFQ’s should also be acknowledged. Further, validation studies for the Nutrition and Health Questionnaire (used in the U.S. sample) have been performed in European samples. Confounding by other dietary or lifestyle factors can also not be ruled out. We have statistically adjusted for a number of variables that related to both predictor (soft drink intake) and outcome (MetS) in both studies, however there may be other unknown factors which impact the relations observed. It also must be acknowledged that the two studies were not conducted at the same period of time, with the MSLS data being collected approximately five years prior to ORISCAV-LUX. One may hypothesise that the availability of diet soft drinks may have increased over time (e.g., more varieties, availability, and accessibility), particularly in Europe. However, our examination of this data actually shows higher consumption of diet drinks in MSLS than in ORISCAV-LUX; whereas the opposite may have been observed if diet consumption was notably higher in 2007-09 than in 2001-06. The time period difference is unlikely to have impacted upon the study findings.

While we are not attempting to generalise beyond the two geographic study sites, the present study does provide insight into how cardiometabolic health differs between the two locations and demonstrates the robust nature of our findings of an association between soft drink consumption and MetS.

## 5. Conclusions

The present study enables us to observe similarities and differences among two culturally diverse samples with regard to drinking habits and health. Despite quite considerable disparities in the two samples examined, in terms of culture and other fundamental parameters, findings were consistent for both studies. This study adds to and supports the growing accumulation of evidence for an association between diet soft drink consumption and MetS prevalence. It has demonstrated that diet soft drinks have an adverse relationship with cardiometabolic health, in two geographically and culturally disparate samples in terms of age, drinking patterns and health status. Randomized-to-treatment, controlled clinical trials to assess how artificial sweeteners consumed from diet beverages impact upon cardiometabolic function are need to confirm observational data obtained in our study. Further research is needed on how the dietary habits in different countries influence health, including the metabolic syndrome, and conversely, how health status influences dietary choice.

## Supplementary Files

Supplementary File 1
