# Supplementary material for: Diet Soft Drink Consumption is Associated with the Metabolic Syndrome: A Two Sample Comparison
Source: Nutrients. 2015 May 13;7(5):3569–86. doi: 10.3390/nu7053569 (PMC4446768; doi:10.3390/nu7053569)
Supplement: Supplementary File 1 [file nutrients-07-03569-s001.docx]

**Supplementary Information**

**Table S1.** Study sample characteristics in ORISCAV-LUX and MSLS studies.

| **Characteristic** | **MSLS, *N* = 803** | **ORISCAV-LUX, *N* = 1323** |
| --- | --- | --- |
| Age (years) | 61.8 ± 12.9 | 44.4 ± 13.1 |
| Sex (% male) | 40.1 | 48.5 |
| Mean no. of soft drinks/day | 0.8 ± 1.4 | 0.5 ± 1.1 |
| Physical activity (mins/day) | 37 ± 49 | 109 ± 134 |
| Smoking (cigs/day) | 1.4 ± 5.4 | 2.8 ± 7.0 |
| MetS (%) | 44.0 | 26.2 |
| Systolic BP (mmHg) | 131 ± 22 | 130 ± 18 |
| Diastolic BP (mmHg) | 70 ± 10 | 82 ± 11 |
| Waist circumference (cm) | 95 ± 15 | 90 ± 14 |
| Total cholesterol (mg/dL) | 202 ± 40 | 202 ± 41 |
| HDL cholesterol (mg/dL) | 54 ± 16 | 62 ± 17 |
| LDL cholesterol (mg/dL) | 121 ± 33 | 124 ± 35 |
| Fasting plasma glucose (mg/dL) | 98 ± 28 | 95 ± 19 |
| Triglycerides (mg/dL) | 144 ±111 | 116 ± 95 |
| BMI (kg/m^2^) | 29.2 ± 6.1 | 26.6 ± 5.0 |
| Diabetes mellitus (%) | 12.0 | 23.3 |
| Hypertension (%) | 61.1 | 39.8 |
| Obesity (%) | 37.8 | 22.6 |
| Dietary variables |  |  |
| Total energy intake ^a^ | 14.5 ± 4.5 | 2418 ± 935 |
| Vegetables (servings/day) | 2.7 ± 1.1 | 3.9 ± 2.7 |
| Fruit (servings/day) | 1.6 ± 1.0 | 1.8 ± 1.9 |
| Grains (servings/day) | 3.6 ± 2.0 | 2.8 ± 1.2 |
| Meat (servings/day) | 2.0 ± 0.9 | 1.2 ± 0.7 |
| Alcohol (standard drinks/day) | 0.5 ± 1.0 | 0.8 ± 0.8 |

Values are mean ± SD unless otherwise indicated; ^a^ Total energy intake: in Kcal/day (ORISCAV-LUX) and total serves/day all food groups (MSLS); BMI = body mass index; BP = blood pressure; HDL = high density lipoprotein; LDL = low density lipoprotein; MetS = metabolic syndrome.

**Table S2.** Comparison of body weight status and soft drink consumption in MSLS and ORISCAV-LUX participants aged 23 to 69 years.

| **Characteristic** | **MSLS, *n* = 565** | | **ORISCAV-LUX, *n* = 1223** | |
| --- | --- | --- | --- | --- |
|  | Mean | SD | Mean | SD |
| BMI (kg/m^2^) | 29.9 | 6.5 | 26.8 | 4.9 |
| Waist circumference (cm) | 95.6 | 15.9 | 90.3 | 13.6 |
| Total soft drinks (serves/day)^a^ | 1.9 | 1.6 | 0.8 | 1.2 |
| Diet soft drinks (serves/day)^a^ | 1.0 | 1.3 | 0.2 | 0.7 |
| Regular soft drinks (serves/day)^a^ | 0.9 | 1.6 | 0.5 | 1.0 |

^a^ In those who consume soft drinks, *n* = 277 in MSLS and *n* = 724 in ORISCAV-LUX;
BMI = body mass index; SD = standard deviation.
